# An Optimized Nature-Inspired Metaheuristic Algorithm for Application Mapping in 2D-NoC

**DOI:** 10.3390/s21155102

**Published:** 2021-07-28

**Authors:** Saleha Sikandar, Naveed Khan Baloch, Fawad Hussain, Waqar Amin, Yousaf Bin Zikria, Heejung Yu

**Affiliations:** 1 Computer Engineering Department, University of Engineering and Technology, Taxila 47050, Pakistan; salu.malik16@gmail.com (S.S.); naveed.khan@uettaxila.edu.pk (N.K.B.); fawad.hussain@uettaxila.edu.pk (F.H.); waqar.ameen45@gmail.com (W.A.); 2 Department of Information and Communication Engineering, Yeungnam University, Gyeongsan 38541, Korea; 3 Department of Electronics and Information Engineering, Korea University, Sejong 30019, Korea

**Keywords:** network-on-chip, sailfish hunting, metaheuristic optimization

## Abstract

Mapping application task graphs on intellectual property (IP) cores into network-on-chip (NoC) is a non-deterministic polynomial-time hard problem. The evolution of network performance mainly depends on an effective and efficient mapping technique and the optimization of performance and cost metrics. These metrics mainly include power, reliability, area, thermal distribution and delay. A state-of-the-art mapping technique for NoC is introduced with the name of sailfish optimization algorithm (SFOA). The proposed algorithm minimizes the power dissipation of NoC via an empirical base applying a shared *k*-nearest neighbor clustering approach, and it gives quicker mapping over six considered standard benchmarks. The experimental results indicate that the proposed techniques outperform other existing nature-inspired metaheuristic approaches, especially in large application task graphs.

## 1. Introduction

The overall performance and scalability of the system-on-chip (SoC) are degraded because of the increasing number of intellectual property (IP) cores embedding on the SoC. For the improvement of overall performance and flexibility of the SoC, new promising solutions have been proposed, and they are called network-on-chip (NoC) [1]. NoC is an on-chip, packet-based communication switching network which is created for interaction between IP cores of the SoC designs [2]. Routers (switch fabric) are linked in some standard topology for communications among IP cores. A router is available for every IP core in an NoC. The router is a basic building block of the NoC architecture; a fault-resilient router architecture is necessary for reliable on-chip communication. The authors of [3,4,5,6] did some architectural modifications in the existing NoC routers designs to propose a reliable on-chip network communication infrastructure. A message passing technique is used for the exchange of data between IP cores. As per the multi-core system principle, the contribution of NoC in power consumption of the total system is around 40%, and this has a vital role in network performance [1,7]. The power, latency and area of NoC-based systems are conspicuously impacted by the selection of an on-chip interconnection architecture [7]. Depending on the interconnection networks, numerous standard topologies are established for the NoC. The most renowned topology out of all prevailing conventional topologies of the NoC architecture is a mesh topology [8].

In the mesh topology, there are short paths for communication between IP cores and high bisection width. The interconnected structure is regular and fixed, and the links are of equal size. Considering this context, various techniques for applications mapping have been proposed using search-based and exact optimization methods. Additionally, proper modeling via an analytical approach has been investigated to reduce the area, latency and power in NoCs.

Because computation time to solve the mapping problem increases with the size of the application to be mapped, it is known that an application mapping is a non-deterministic polynomial-time (NP)-hard problem. To obtain the optimal solution over NoC performance metrics, search-based optimization techniques have been considered. Therefore, the solution of NP-hard problems is significantly dependent on the choice of the best heuristic or metaheuristic technique.

In practical systems, resources are limited so that an efficient utilization of given resources is a critical issue. Optimization techniques can be employed in a wide range of areas, including engineering, finance, resource planning and Internet routing. Using a mathematical model of the social and political progression, metaheuristic algorithms provide an effective algorithm to solve the given optimization problems. These algorithms can obtain a universal solution by facilitating interaction between high level approaches and local improvement methods.

Furthermore, a metaheuristic algorithm can be efficient if it offers a realistic equilibrium between experimentation and exploitation on a provided optimization problem, which is critical. Intensification (i.e., exploitation) is associated with local search, while diversification (i.e., exploration) is associated with global search. Diversification tends to find out diverse solutions globally (i.e., global search). On the other hand, intensification focuses on searching local regions with the knowledge of the current best solution from this region (i.e., local search). There is no initial solution required for global search, while local search starts from an initial candidate solution. The mobility of candidate solutions should be randomized as far as possible during the exploration phase. On the other hand, the exploitation process entails thorough investigation of the promising area(s). The most dominant difference between current metaheuristic algorithms, in general, is how they balance the discovery and exploitation phases. Depending upon the context mentioned above, sailfish optimization (SFO) is considered in this study.

SFO provides a suitable equilibrium between intensification (exploitation) and diversification (exploration) to avoid early convergence. To examine the performance metrics of NoC, the novel metaheuristic optimization algorithm used in this paper, that is, SFO, is described in [9]. The SFO algorithm is modeled after a sailfish group targeting a school of sardine prey in a series of attacks. To begin, SFO uses two assortments of prey and predator species to replicate the technique of group hunting. Second, the presented algorithm breaks down the mutual security of grouping prey by alternating attacks. Third, prey mobility can be changed across the search region, allowing the hunter to capture the right prey and improve its fitness. The effectiveness of the SFO algorithm is verified by examining the optimal mapping for eight NoC benchmarks for the two-dimensional (2D) mesh topology.

The remainder of the paper is structured as follows. The related work is given in Section 2. The inspiration for the sailfish optimization algorithm is described in Section 3. The mapping using SFO, models used for the analysis of metrics and the proposed algorithm are described in Section 4, Section 5 and Section 6, respectively. The experimental setup along with considered benchmarks and results are summarized and analyzed in Section 7. Section 8 ends with some conclusive remarks.

## 2. Related Work

In [10], Araki and Yoshihiro presented a multi-path reliable distance-vector routing strategy by utilizing multiple paths for the extension of reliable distance-vector routing (RDV) for the improvement of communication performance, decreased delivery delay, higher load-balancing and more substantial network capacity. In comparison to RDV, fault tolerance is also greater against the topology modifications. In [4], Rashid et al. proposed a reliable on-chip network communication architecture by making some architectural improvements in the existing NoC routers’ designs. In [11], a router’s controllers design based on finite-state machine (FSM) is presented for the minimization of error propagation, aiming at low utilization of logical resources.

In [12], Wu and Cai presented a Fibonacci tree optimization strategy (FTOS) for the scheduling query of wireless sensor networks. The proposed algorithm provided less energy consumption and optimization of detection efficiency. In [13], Rhee et al. presented an artificial neural network (ANN) model combined with the genetic algorithm (GA) for the cost-effective operation of a silo. The combined technique gave the optimized results with the improvement in the accuracy of internal level prediction of the silo, and an efficient number of sensors and their positions of installation are determined. In [14], the authors presented a comprehensive overview of the algorithms of machine learning for embedded systems and mobile computing space. In [15], the authors presented a heuristic technique based on the moth-flame optimization (MFO) algorithm for resolving the weak exploration problem of the k-means data clustering algorithm.

The problem of application mapping has stimulated the research community because of the expeditious growth in NoC. Tosun et al. proposed integer linear programming (ILP) as an exact mapping method for the mesh-based two-dimensional NoC with an energy minimization principle in [8]. In [16], Hu and Marculescu presented a branch and bound (BB) mapping solution for the topological allocation of IP cores on an NoC platform for the minimization of the total consumption of energy with the limitation of bandwidth of the link. In [17], Lei et al. presented a two-step genetic algorithm (GA) based on delay for the communication of NoC. The prime function for the scheduling and mapping of IPs was the minimization of overall execution time. Murali and Micheli proposed a heuristic approach based on a mapping algorithm for cores mapping on 2D mesh topology with the restraint of bandwidth reservation in [18]. In [19], Lu et al. presented a clustering algorithm based on simulated annealing for reducing the simulation time of an annealing process of a large system. The process of clustering compromised the optimum results but accelerated the computation time. In [20], Radu and Vintan proposed an optimized simulated annealing (OSA) algorithm for 2D mesh mapping by optimizing the parameters of the annealing process for producing the optimum outcomes with less time than the conventional simulated annealing schemes. Ascia et al. [21] presented a multi-objective GA for mapping of IP cores in a 2D mesh topology for optimizing the power consumption and network performance. In [22], Jena and Sharma presented a heuristics search based multi-objective GA for the mapping of IP cores on a 2D mesh topology for the optimization of link bandwidth, the performance of the network and power dissipation. Sepulvada et al. also presented a multi-objective adaptive immune algorithm (MAIA) for the problem of application mapping of NoC architecture [23]. In [24], Harmanani and Farah proposed an algorithm for assigning tasks to the nodes of a 2D mesh network based on simulated annealing. Hu et al. proposed a task mapping technique for the NoC architecture with a constraint of bandwidth [25]. This technique was energy aware and expedited the run-time of the process of task mapping, but it shows trade-off in the network performance results.

Ye et al. derived the power models for connectivity wires, switch and inbuilt buffer in [26]. In [27], the authors provided a well-accepted mathematical term for 2D NoC interconnect energy models. Kahng et al. [28] and Ost et al. [29] created a practical power model for 2D NoC as a follow-up to the one in [27]. In [28], the power model takes into account architecture-level power as well as region modeling and router capacity for the router. The power modeling in [28] was validated and checked by Ost et al. [29]. The authors of [30] calculated the efficiency of mesh-dependent 2D and 3D NoCs based on the comprehension of energy depletion between the cores and the routing area. The thesis by Sahu and Chattopadhyay [31] takes advantage of a comprehensive review of framework mapping techniques for NoC and examines various mapping methods proposed during the last period. As per Sahu and Chattopadhyay [31], a heuristic-based mapping strategy provided a better end result in terms of network output metrics optimization.

In [32], a simulated annealing (SA) algorithm is implemented as a metaheuristic approach to create an efficient mapping with IP connectivity specifications as a restriction for 2D NoC. The authors of [33] implemented mapping by scheduling with an ant colony optimization (ACO) approach for 2D NoC. In [34], a particle swarm optimization (PSO) is used as a mapping technique on both 2D and 3D NoCs, with the connectivity metric as the objective function. To tackle the problem addressed in [32], a power-aware mapping technique for 2D NoC utilizing SA with the taboo quest (SAT) was proposed by Alagarsamy and Gopalakrishnan [35]. In [36], a mapping technique for a 2D NoC is presented. The foremost objective is to build a chain of linked cores that can be used to construct a new mapping system. In comparison to similar ones, the authors of [36] attempted to use less bandwidth. In [37], Tosun presented a heuristic approach for a mesh 2D NoC in which a priority list based on overall and average communication bandwidth was established.

In [38], a reliability-aware technique is presented. The featured graph is divided into two sub-graphs, which are used to reduce transmission flow. As a result, transmission flow between the two sub-graphs is reduced, while traffic within every graph increases. Niknam and Amiri presented a novel hybrid PSO-based approach to address the clustering issue in [39]. For better performance, ACO and *k*-means techniques were used. The presented approach was tested and validated on various publicly available datasets, and the preliminary observations are optimistic. The suggested hybrid approach was shown to coincide with an optimal solution in the majority of instances. Junior et al. [40] also presented an ACO-based approach for finding and maximizing directions in a mesh-based NoC. Routed optimization was achieved by reducing the total delay in packet transmission between activities. The visionary conclusions showed the efficiency of the ACO-based technique. In addition, Xie et al. proposed an online mapping protocol to refine task mapping methodology for minimizing connection power consumption [41]. First, the run-time interconnection point of applications was investigated. Secondly, this method measured the mapping assignment and used real-time web mapping.

## 3. Sailfish Optimizer

In this section, the key inspiration for the SFO algorithm (SFOA) is discussed. The suggested algorithm and mathematical models are then thoroughly explained.

### Inspiration

Shadravan et al. [9] recently introduced a new metaheuristic technique called SFO, which incorporates the action of both a predatory group of sailfish and a prey group of sardines. The sailfish is known as a social predator since it attacks and catches its prey in groups. Predators use various killing techniques in cooperative hunting. The class of sailfish, for example, is distinguished by the alternation of attack techniques. It entails that each member of the group attacks the school of prey (sardine) alone at a given time, injuring or hunting some of them while the other group members conserve their strength. Whenever a sailfish attacks a school of prey, it will update its location concerning them. Furthermore, the sailfish will update their location to occupy vacant space around the prey school and imitate circling the prey. When a member of the sardine group (prey) is wounded, the sardine group changes direction to avoid the sailfish’s subsequent attacks. The general procedure of the sailfish optimizer algorithm is defined in the subsections that follow.

Group hunting is an intriguing illustration of collective activity in communities of invertebrates, fishes, birds and mammals. Compared to hunting alone, predators do not require a lot of power to kill their prey while hunting in groups.

Predators in the most basic type of group hunting aim to finish off the prey by step-by-step planning of the attack, whereas predators under the more sophisticated class of group hunting practice specialized positions to mob and capture the prey [42]. The alternation of attacks is one of the most complicated group hunting techniques. This tactic allows the hunter to save strength when other predators are injuring the prey. Sailfish hunting in groups that alternate attacks on the schooling sardines is an illustration of this kind of method [43,44].

The most expeditious fish in the ocean, sailfish can attain speeds up to 62 miles per hour. They hunt in clusters, herding schools of smaller fish, such as sardines, near the surface. Sailfish find the sardines’ mobility and speed during the assault very difficult. The sailfish either slashes multiple sardines with its rostrum or taps a single sardine, causing it to become unstable. Sardines cannot float quickly enough to dodge the tip of the sailfish’s rostrum and are incapable of responding to this community hunting because the sailfish has one of the fastest accelerations ever observed in a floating creature. According to sardine experimental action, wounded sardines would be isolated from the prey shoal and unable to travel with the shoal, resulting in their capture by the sailfish [42].

The majority of sailfish attacks do not result in sardine deaths, and only a small percentage of sardines are directly caught. However, as sailfish attacks become more common, an increasing number of sardines are injured. Animals who hunt in groups, such as wolves, are more likely to engage in this form of hunting. On the other hand, these sailfish parties split up and regroup with new affiliates daily. During an assault, a sailfish preserves its big back flipper and sacral flippers upright to maintain its body strength. Often, right before an attack, they transform their body color from the usually bluish-silver parallel edges deepening to nearly black. The purpose for the color change is unclear, but it appears to be a form of communication between sailfish [42]. Sailfish use shifts in their body to signal which should move first, allowing them to avoid being injured by a companion. The attack-alternation technique of sailfish party hunting is the key inspiration for the SFO algorithm. The natural actions of sailfish and sardines are mathematically represented in the following subsection, and an optimization approach based on this mathematical model is developed.

## 4. Mapping Using SFOA

### 4.1. Problem Formulation

An application is characterized by a directed graph of the network in NoC, which is later scheduled by the scheduler using another directed core graph of the network on the existing IP-cores. The directed core graph is transmuted and depicted via an effective mapping method on the NoC topological architecture using an architecture graph.

**Definition 1.**  ***Directed Task Graph (DTG):** The task graph of the network is a directed acyclic graph DTG(P, E), where every node of the graph symbolizes a task of the computational process of the application. In addition, the directed edges or links represent the communication or data volume among the tasks communicating*.

(1) DTG(P,E)

where *P* and *E* are the sets of nodes, which correspond to the processes or tasks, and links or edges, respectively, and $p_{i}\in P$, $e_{i,j}\in E$ for i,j=1,2,3,⋯.

**Definition 2.**  ***Directed Core Graph (DCG):** The core graph of the NoC architecture is a directed graph DCG(C, D), where every node of the graph symbolizes the IP cores in the topology. The directed edges represents the direct communication among the nodes (i.e., IP cores, d_i_ and d_j_)*.

(2) DCG(C,D)

where *C* is the set of IP cores or processing elements and *D* denotes the set of links or edges with communication directions in the architecture graph. Elements in *C* and *D* are defined as $c_{i}\in C$ and $d_{i,j}\in D$ for i,j=1,2,3,⋯.

### 4.2. SFOA for NoC Mapping

The initial sailfish and sardine populations are generated using the initial mapping and weight of the task graph given at time t=0. Considering the settings of parameters of the proposed algorithm, the fitness value, which is the communication cost (CC) of the best sailfish (i.e., mapping solution), is computed. (For CC, refer to Equation (8) which is defined in Section 5). Later, the positions of sailfish and sardine are updated in in consideration of attack power (AP). (For position updates of sailfish and sardine, refer to Equations (21) and (27), respectively. For AP, refer to Equation (24) in Section 5). After updating the positions, the optimized result of mapping (sailfish) can be obtained.

### 4.3. Parameters Setting for SFOA

The proposed algorithm requires the setting of a few basic parameters to verify the efficiency of group hunting. In the proposed algorithm, the fitness function under consideration is the cost for communication, which is denoted by CC. The population size is 300, the number of iteration is equal to 150 and pp is the rate between the sailfish and sardine (where pp is defined as the fraction of the sardine population which forms the initial sailfish population), which is set to 0.1; these values are set for the application mapping on 2D NoC. These values are set based on the number of iterations run and optimization acquired for deducing an optimal solution. They also differ as per the properties of the application considered for mapping.

For the analysis of the performance parameters of an NoC such as energy, power and communication cost computation along with latency and average throughput, two models are used in this work. These two models are named the Bit Energy model and CMOS cell library model, and their mathematical expressions are explained in detail and in the next section.

## 5. Models Used for Analysis of Metrics

For analyzing the performance metrics of an NoC, two models are considered in the presented work [25,43]. An effective trade-off between the faster mapping over 2D mesh and performance metrics of NoC is presented by SFOA in this study.

### 5.1. Bit Energy Model

For the estimation of consumption of power of the router in the network, an energy model [25] is considered as follows:

(3) $$E_{B}=E_{SB}+E_{LB},$$

where $E_{B}$ is the energy used up for transferring 1 bit of data from the source node to the destination node, which comprises the energy of the switch ($E_{SB})$ and energy of the link ($E_{LB}$) of the NoC network. The average network energy consumption $E_{B(p_{i},p_{j})}$ for transferring 1 bit of data from a source node $p_{i}$ to the destination node $p_{j}$ is calculated by the following equation:

(4) $$E_{B(p_{i},p_{j})}=H_{count}\times E_{SB}+{(\;H}_{count}-1)\times E_{LB},$$

where $H_{count}$ is the Manhattan distance between the source node $\left(a_{i},a_{j}\right)$ and the destination node $\left(b_{i},b_{j}\right)$, which is obtained by

(5) $$H_{count}=\left|a_{i}-b_{i}\right|+\left|a_{j}-b_{j}\right|.$$

Therefore, the total energy consumption of the network *(E_T_)* is calculated by using the average network energy and the link bandwidth, $BW_{\left(p_{i},p_{j}\right)}$, between nodes $p_{i}$ and $p_{j}$.

(6) $$E_{T}=\sum_{i,j}\left(E_{B(p_{i},p_{j})}\times BW_{\left(p_{i},p_{j}\right)}\right)$$

Substituting Equation (4) into Equation (6), $E_{T}$ can be rewritten by

(7) $$E_{T}=\sum_{i,j}\left[\left(H_{count}\times E_{SB}+\left(H_{count}-1\right)\times E_{LB}\right)\times {BW}_{\left(p_{i},p_{j}\right)}\right].$$

Moreover, the cost of communication is defined by

(8) $$CC=\sum_{i,j}H_{count}\times {BW}_{\left(p_{i},p_{j}\right)}.$$

Different mapping results generate different energy and cost values. The prime concern is to obtain a mapping function that provides minimal cost for the whole network. The communication cost of the applications of NoC is considered the performance measure for distinct applications in this research work.

### 5.2. CMOS Cell Library Model

The proposed SFO algorithm utilizes the standard CMOS cell library model [43] for the calculation of network power, latency, energy consumption of packets and throughput of an NoC system. For the computation of average latency of the network via this model, the following equation is used:

(9) $${Lat}_{avg}=\frac{1}{N}\sum_{i=1}^{N}\left(\frac{1}{N_{i}}\sum_{k=1}^{N_{i}}\left({Lat}_{\left(i,j\right)}\right)\right),$$

where *N* is the total number of processor or cores in the network, $N_{i}$ is the total numbers of received packets by the core *i* and $Lat_{\left(i,j\right)}$ is the latency of packet *j* at destination node *i*.

The average throughput of the network, $TP_{avg}$, is evaluated as follows:

(10) $$TP_{avg}=\frac{1}{N(T_{S}-T_{W})}\sum_{i=1}^{N}N_{i},$$

where $T_{W}$ is the warm-up time of the simulation and $T_{S}$ is the simulation time.

The network average power, $P_{Navg}$, is computed by

(11) $$P_{Navg}=\frac{1}{N}\sum_{i=1}^{N}\;\sum_{k=1}^{N_{i}}\;\left[\alpha_{\left(i,k\right)}P_{N(act,k)}+\left(1-\alpha_{\left(i,k\right)}\right)P_{N(inact,k)}\right]$$

where $\alpha_{\left(i,k\right)}$ is the active probability of component *k* in router *i* after $T_{W}$. Moreover, $P_{N(act,k)}$ and $P_{N(inact,k)}$ are the post-layout active and inactive power of the component *k*.

Finally, the network average energy consumption by every packet is given by

(12) $$E_{Pavg}=\frac{T_{S}-T_{W}}{NN_{pack}}\sum_{i=1}^{N}\;\sum_{k=1}^{N_{i}}\;\left[\alpha_{\left(i,k\right)}P_{N(act,k)}+\left(1-\alpha_{\left(i,k\right)}\right)P_{N(inact,k)}\right]$$

where *N* is the total number of cores available in the network. $N_{pack}\left(=\sum_{i=1}^{N}N_{i}\right)$ is the total number of packets injected in the network. For a certain number of experiments, *N* remains the same, and $N_{pack}$ can be changed by increasing or decreasing the packet injection rate.

## 6. The Proposed Algorithm: SFOA

The proposed SFOA takes the inputs, directed task graph, DCG, and directed network graph, DNG, and effectively performs the mapping of the task onto the cores of the 2D NoC topological architecture.

### 6.1. Empirical Base for Initial Mapping

**Figure 1:**
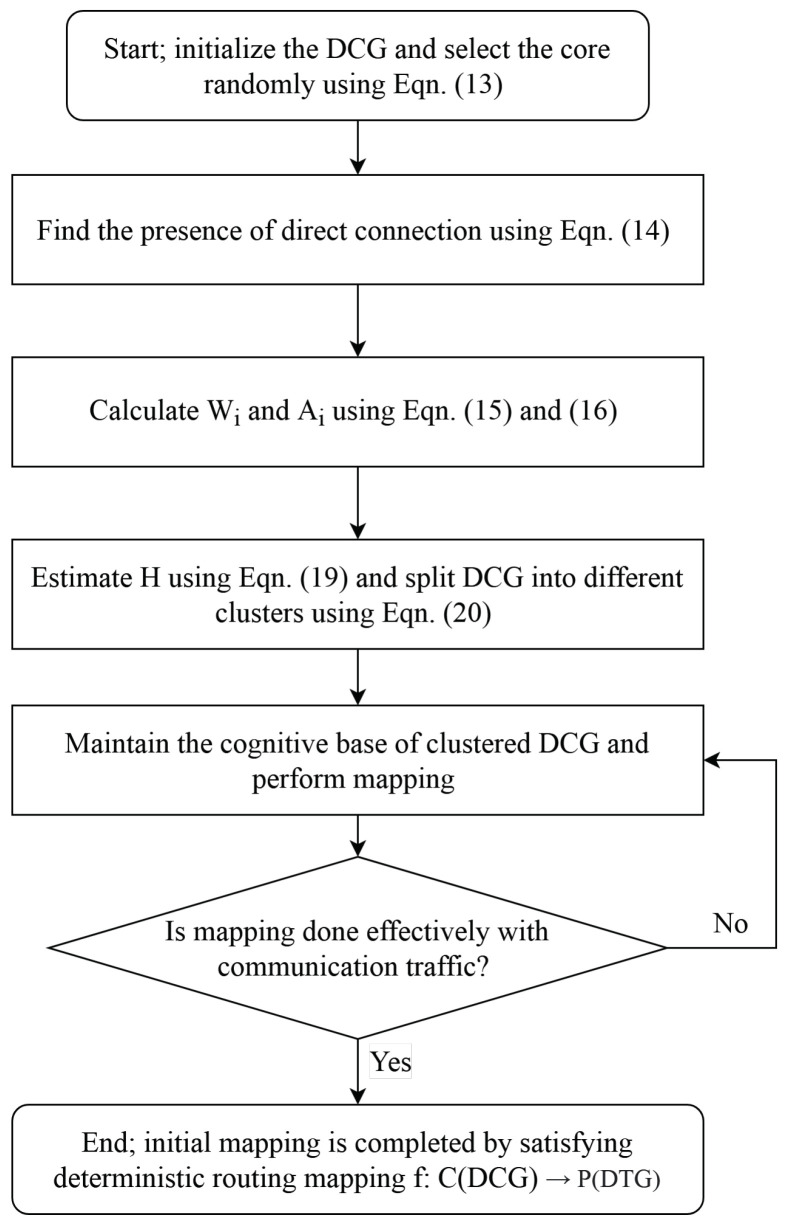
Flowchart for initial mapping.

To create the empirical base for the initial mapping, the following five steps of the self-adaptive chicken swarm optimization (SCSO) algorithm [44] are considered. Furthermore, Figure 1 shows the flowchart for initial mapping procedure.

- Step 1: From DCG, randomly select the IP-Core
  (13) $$Rand\left(c_{i}\right),\;\;\mathrm{for}\;{\;\;c}_{i}\in C$$
- Step 2: Use the DC matrix to find the presence of direct connection of the selected core with each core.
  (14) $$DC=\left\{\begin{array}{c} \;1\;\;\;\;\;\;\;\;\;\;;\;\;\;\mathrm{if}\;\left(c_{i},c_{j}\right)=d_{ij}\in D\\ \;0\;\;\;\;\;\;\;\;\;\;;\;\;\;\;\;\;\;\;\;\;\;\;\;\;otherwise \end{array}\right.$$
- Step 3: Calculate the average CC ($A_{i}$) and weight ($W_{i}$) for each core ($c_{i}$) as follows:

(15) $$W_{i}=\sum_{d_{ij}\in D}w_{ij}$$

(16) $$A_{i}=\sum_{d_{ij}\in D}\frac{w_{ij}}{\left|N\left(c_{i}\right)\right|},$$

where $w_{ij}$ is the weight between cores $c_{i}$ and $c_{j}$ and $N(c_{i})$ is the open neighborhood of $c_{i}$.

For the identification of neighbors, use the following equation:

(17) Nci=ci∈Cci∈Cci,cj=dij∈Dci,cj=dij∈D

- Step 4: For the identification of hop counts among the source node $c_{i}$ and sink node $c_{j}$, use the following matrix:

(18) $$H=\left[H_{ij}\right]$$

where $\left[H_{ij}\right]$ means that (i,j) element of matrix *H* is given by $H_{ij}$. Matrix *H* indicates the minimum probable links for communication between the source and sink nodes. Considering $d(c_{i},c_{j})$ is the shortest path between the cores $c_{i}$ and $c_{j}$, $N(c_{i},c_{j})$ is the number of hops in the shortest path.

(19) $$H_{ij}=\min\left(N\left(c_{i},c_{j}\right)\right)$$

- Step 5: Using the shared *K*-nearest neighbor clustering approach, form a diverse cluster. If $c_{i}$ and $c_{j}$ have each other in their closest *K*-nearest neighbors list, then an edge exists between them. The strength of this edge is evaluated using:

(20) $$str\left(c_{i},c_{j}\right)=\sum \left(K+1-o\right)\times \left(K+1-p\right)\;\;$$

where *K* is the size of the neighbor’s list, *o* is the position of shared near-neighbor in $c_{i}$ list and *p* is the position of shared near-neighbor in $c_{j}$ list. Hence, $c_{io}$, i.e., the shared near-neighbor in $c_{i}$ list, is equal to $c_{jp}$, that is the shared near-neighbor in $c_{j}$ list.

**Figure 2:**
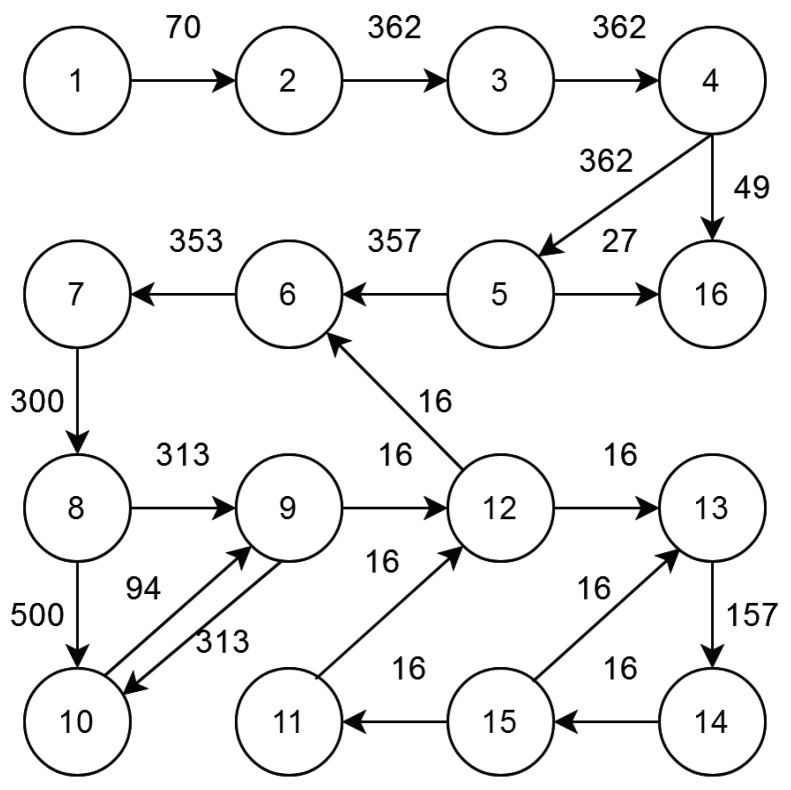
Standard NoC VOPD benchmark.

**Figure 3:**
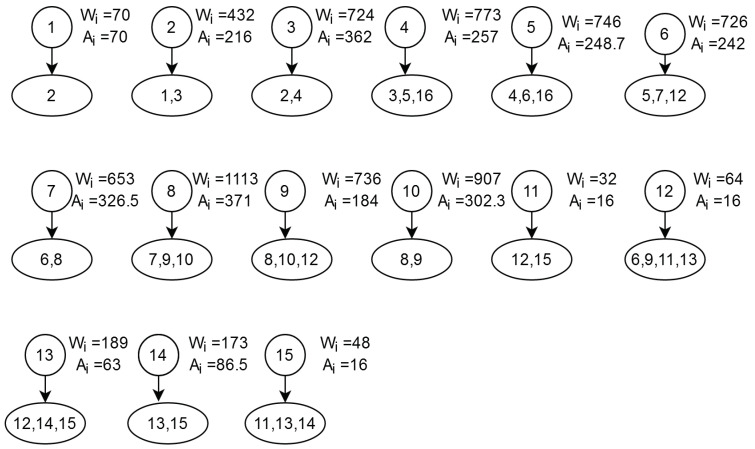
Clustering of VOPD task graph.

**Figure 4:**
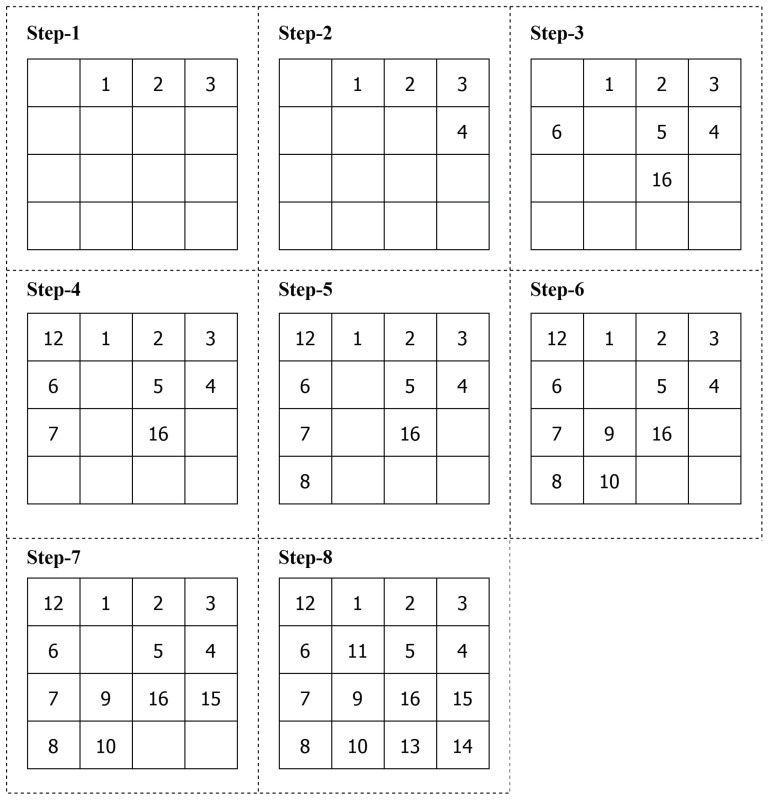
Initial mapping of VOPD task graph on 4×4 mesh.

After Step 5, an empirical base is created with clustered DCG. Figure 2, Figure 3 and Figure 4 show the standard NoC video object plane decoder (VOPD) benchmark, clustering of VOPD task graph and its initial mapping on a 4×4 mesh, respectively.

### 6.2. Video Object Plane Decoder

**Figure 5:**
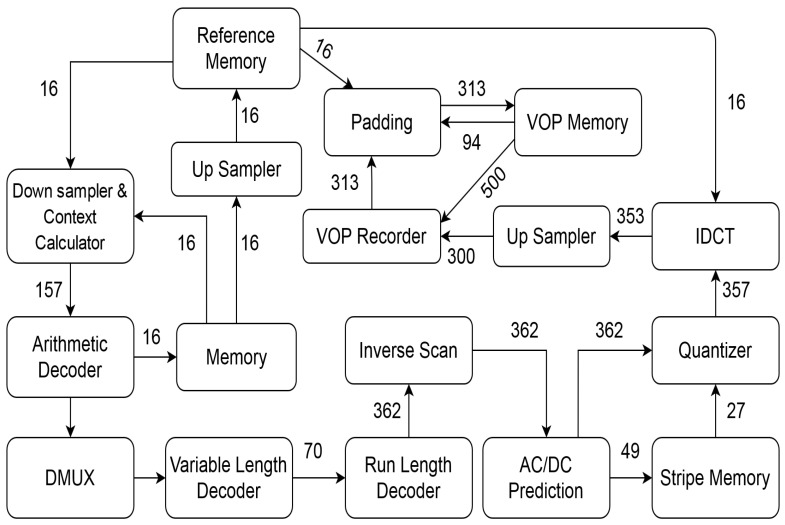
Architectural representation of VOPD.

Video object plane decoder (VOPD) is an application comprising several sub-tasks: run-length decoder, downsampler, quantizer, etc. These sub-tasks require communication among themselves at the rates specified in MBs on the edges between them. Figure 5 represents the architectural diagram of the VOPD, while Figure 2 illustrates the graphical representation of the VOPD tasks. VOPD consists of 16 sub-tasks having 21 edges labeled with distinct communication bandwidth.

**Figure 6:**
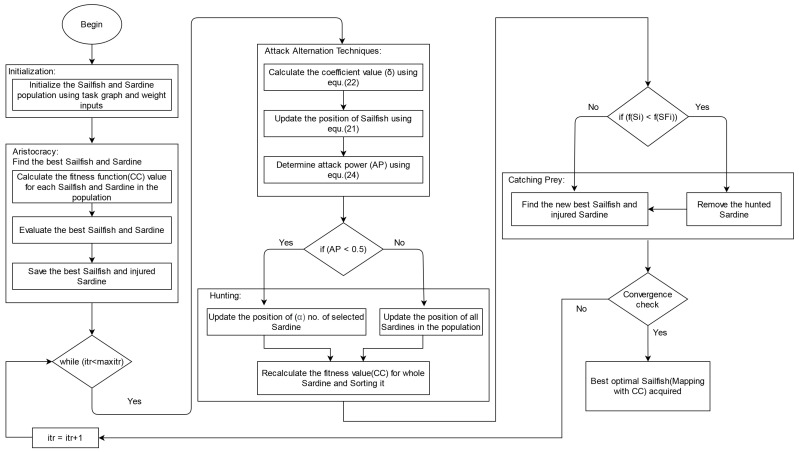
Flowchart for proposed SFOA.

For the initial phase of mapping, a random procedure is adopted as a mapping strategy. The outcome of this initial mapping is considered the input for the proposed SFOA to minimize the consumption of power and communication cost of 2D NoC. Figure 6 represents the flowchart for SFOA.

### 6.3. SFOA Algorithm

#### 6.3.1. Initialization

The first step of SFOA comprises initialization of the sailfish and sardine populations. The population generation/initialization is random. Variable position vectors represent that the sailfish can search in multiple dimensions. In this algorithm, the candidate solution considered is sailfish and the positions of sailfish in the search space are the variables of the problem. Firstly, the sailfish and sardine populations are randomly initialized as ${XSF}_{i}^{itr}$ and ${XS}_{j}^{itr}$, which are the position of sailfish and sardine populations where the subscripts *i* and *j* are the indices of sailfish and sardine from the initialized population and the superscript itr denotes the index of iteration.

#### 6.3.2. Aristocracy

A sailfish hunts the sardine while exploring the search region and updating its location/position to find a better solution. While updating the location of the sailfish, which is the search agent in this algorithm, better solutions may be lost. There is the possibility that the updated positions can be worse than the previous positions, thus elitism/aristocracy is applied.

Aristocracy involves finding the best search agent via the best sailfish fitness value and, for the sardines, the best fitness value of injured sardine and replicating the unchanged best solutions to the next generation. The best position of the search agent (sailfish) is kept in every iteration and measured as an Elite. The best or the fittest sailfish acquired until now is the Elite sailfish. It would be the one affecting the maneuverability and speeding up of sardines during the attacking. The location of any injured sardine is also saved in every iteration, which the sailfish will consider for group hunting as the best target selected.

Secondly, the fitness of each sailfish and sardine in the population is calculated using the fitness function (i.e., CC in the proposed algorithm). Based on this, Elite (i.e., the best sailfish) and injured sardine are acquired. The best sailfish is the one having the smallest fitness function value at iteration itr.

$$XSF_{best}^{itr}=\left\{XSF^{itr}|\mathrm{sailfish with the smallest fitness value}\right\}$$

Similarly, the injured sardine is the one which has been attacked and injured by the sailfish and having the smallest value of CC.

$$XS_{inj}^{itr}=\left\{XS^{itr}|\mathrm{sardine injured by the best sailfish}\right\}$$

#### 6.3.3. Attack-Alternation Technique

Sailfish promote the success rate of hunting their prey with the help of attacking in coordination technique. Sailfish chase their prey and herd them, change their own position conferring to the position of the other hunting sailfish, without even directly communicating with each other. Through this attack-alternation technique, sailfish injure more sardines during the first phase of hunting, which leads to a higher rate of success in capturing the prey at advanced phases of group hunting.

Afterward, the termination condition is checked. If the condition is not satisfied, the position of sailfish is updated with the following equation:

(21) $${XSF}_{new}^{itr}={XSF}_{best}^{itr}-\delta_{itr}\times \left(\varphi \times \left(\frac{{XSF}_{best}^{itr}+{XS}_{inj}^{itr}}{2}\right)-{XSF}_{old}^{itr}\right).$$

The symbols in the above update equation are defined as follows: ${XSF}_{new}^{itr}$ is the updated position of sailfish, ${XSF}_{best}^{itr}$ is the position of best sailfish, $\delta_{itr}$ is the coefficient at iteration itr, φ is a random number between 0 and 1, ${XS}_{inj}^{itr}$ is the position of injured sardine and ${XSF}_{old}^{itr}$ is the current position of sailfish.

(22) $$\delta_{itr}=2\times \varphi \times P.$$

where *P* denotes the prey density.

The prey density represents the quantity of prey at each iteration. It is an important factor when updating the position of sailfish because the number of prey (i.e., sardines) will decline in group hunting as follows:

(23) $$P=1-\left(\frac{nSF}{nSF+nS}\right)$$

where nSF and nS denote the numbers of sailfish and sardine, respectively, in each iteration.

After using Equation (21) for updating the position of sailfish, the attacked power of sailfish, AP, at iteration itr is calculated with

(24) $$AP=C\times \left(1-\left(2\times itr\times \epsilon \right)\right),$$

where *C* and epsilon are the coefficients for linearly decreasing *AP*.

#### 6.3.4. Hunting Prey

The observation of a complete massacre of sardine is very sporadic at the beginning of the group hunting. In more than 90% of the cases, the scales of sardines would be removed after the sailfish strikes their bodies. At the start of the hunting phase, the energy level of sailfish for hunting and catching its prey is higher, and the sardines are also not really drained and injured. This is the reason that sardines have excessive escape speed and high maneuverability. Sailfish’s attacking power would decline steadily over the time of hunting.

The position of every sardine in the population is also updated based on the current position of sailfish and AP at every iteration. The following formula is used for updating the position of sardine:

(25) $${XS}_{new}^{itr}=\varphi_{1}\times \left({XSF}_{best}^{itr}-{XS}_{old}^{itr}+AP\right),$$

where ${XS}_{new}^{itr}$ and ${XS}_{old}^{itr}$ are the updated and previous positions of sardine and $\varphi_{1}\;$ is a random number between 0 and 1.

Considering the value of AP, if the attack power of sailfish is less than 0.5, only *S* number of sardines positions will be updated. Otherwise, all the sardines’ positions will be updated. Here, *S* is determined by

(26) S=nS×AP.

Next, the fitness value (i.e., CC) of all the sardines and sailfish is recalculated as per their updated positions and population is sorted.

#### 6.3.5. Catching Prey

Alongside the reducing attacking power of sailfish, the energy levels of sardines would be decremented because of the recurrent powerful attacking of sailfish. The attacks also affect the maneuverability as it reduces the prey’s ability to detect the directional information regarding the position of sailfish. This will result in pulling away the sardines from the school after being slashed by the sailfish’s rostrum, and they would be quickly captured then.

In the last phase of hunting, the pulled away sardines are quickly captured by the sailfish. In this algorithm, it is considered that, if any sardine becomes fitter than the sailfish, it is removed from its population. The sailfish will update to the position of the corresponding sardine as follows:

(27) $${XSF}^{itr}={\;XS}^{itr},\;\;if\;\;CC\left(S_{itr}\right)<CC\left({SF}_{itr}\right),$$

where $CC\left(S_{itr}\right)$ and $CC\left({SF}_{itr}\right)$ denote the fitness values (i.e., CC values) of sardines and sailfish at iteration itr.

Thereafter, the position of best sailfish and injured sardine is also updated at every iteration.

#### 6.3.6. Deducing Optimal Sailfish

The injured sardine that pulled away from the school would quickly be captured. In SFOA, it is considered that, when a sardine becomes weak, its respective sailfish catches its prey. The hunted sardine’s position replaces the sailfish’s position, elevating the probabilities of new prey’s hunting. After satisfying the termination condition, the best sailfish is acquired along with its fitness value, that is CC.

## 7. Results and Discussion

This section presents the results of the performance analysis of SFOA for 2D NoC for six standard NoC benchmarks, as shown in Table 1. Network size is standard 4 × 4 for all considered benchmarks. For a fair comparison with previous state-of-the-art architectures, the network size is the same. VOPD application consists of 16 sub-tasks. These sub-tasks can be mapped on a 4 × 4 mesh network. However, in the case of MPEG4, MWD, MP3encMP3dec, 263encMP3dec and 263decMP3dec 4, 4, 3, 4 and 1 routers are idle, respectively.

### 7.1. Experimental Setup

To evaluate the performance of the proposed SFOA, different standard NoC benchmarks were considered and various experiments were conducted. The proposed algorithm was verified for 2D NoC with other nature-inspired algorithms such as ACO, PSO, GA, SA and CSO. The code for the proposed SFOA algorithm was written in Python and implemented on NoC Tweak Simulator [43]. All experiments were run on a PC Intel(R) Core (TM) i7-16GB RAM, 2.30 GHz processor. Table 2 depicts the details of the NoC Tweak platform for simulation.

### 7.2. Average Power Dissipation Analysis

To evaluate the efficiency of the proposed algorithm, power minimization analysis was also performed. It shows that SFOA outperformed other existing mapping techniques and the average percentage of improvement on power minimization with other nature-inspired algorithms.

Table 3 shows the results for total power consumption in watts (W) of 2D 4×4 mesh for six standard NoC benchmarks. From the results in Table 3, it is evident the average improvement of power minimization of our proposed algorithm SFOA is 3.63%, 23.7%, 18.70%, 22.14%, 27.25%, 18.66%, 12.08% and 4.73% over ILP, ACO, PSO, SAT, SA, GA, BA and CSO, respectively.

### 7.3. Communication Cost and Computation Time Analysis

The execution analysis of the proposed SFOA compared to other present nature-inspired mapping algorithms is presented in this section. Table 4 depicts the evaluation of average communication cost $\left(H_{count}\times BW\right)$ from Equation (8) for VOPD [34] and MPEG4 [30] standard NoC benchmarks for two-dimensional NoC.

As ILP [8] is regarded as one of the most competent algorithms in the exact mapping method for communication cost estimation, our proposed SFOA esd explicitly compared with ILP as well, along with other algorithms. SFOA provides the same results for communication cost, as shown by the results in Table 4. The values of a few parameters are missing in Table 4, Table 5 and Table 6 for some benchmarks as they were not provided by the authors in the base papers of ACO [33] and SA [32].

The percentage deviation from the exact mapping method based on ILP over heuristic-based mapping techniques for 2D NoC is shown in Table 5. However, the proposed SFOA gives the best results compared with other nature-inspired algorithms, as specified by the results in Table 6. In comparison with other existing mapping techniques, the proposed SFOA takes 69% less computation time. Table 6 represents the estimations for computation time in seconds and communication cost in MB/s of two-dimensional 4×4 mesh for six standard NoC benchmarks.

### 7.4. Average Network Latency Analysis

For the analysis of the performance of the proposed SFOA, the impact of average network latency was also scrutinized with different types of traffic patterns on mesh topological architecture. The considered distinct types of traffic patterns are a uniform random traffic pattern and tornado traffic pattern. These traffic patterns are a method for defining the communication between the IP-cores of the NoC.

In the case of uniform random traffic patterns, it distributes the traffic uniformly, balances the load and each source is equally likely to communicate with each destination. In the case of tornado traffic patterns, it is devised as a combatant for torus topologies.

The performance analysis of the considered 4×4 mesh-based NoC architecture was done using the XY-routing algorithm via the NoCTweak simulator [43]. The average network latency of the proposed algorithm, i.e., SFOA, was evaluated for the above-considered two types of traffic patterns compared with other existing nature-inspired heuristics algorithms.

**Figure 7:**
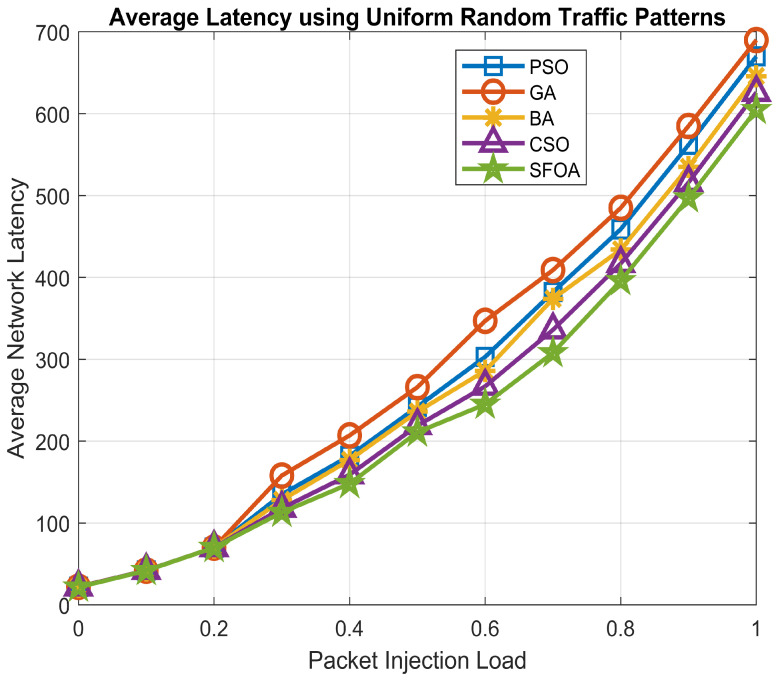
Average network latency for uniform random traffic patterns.

**Figure 8:**
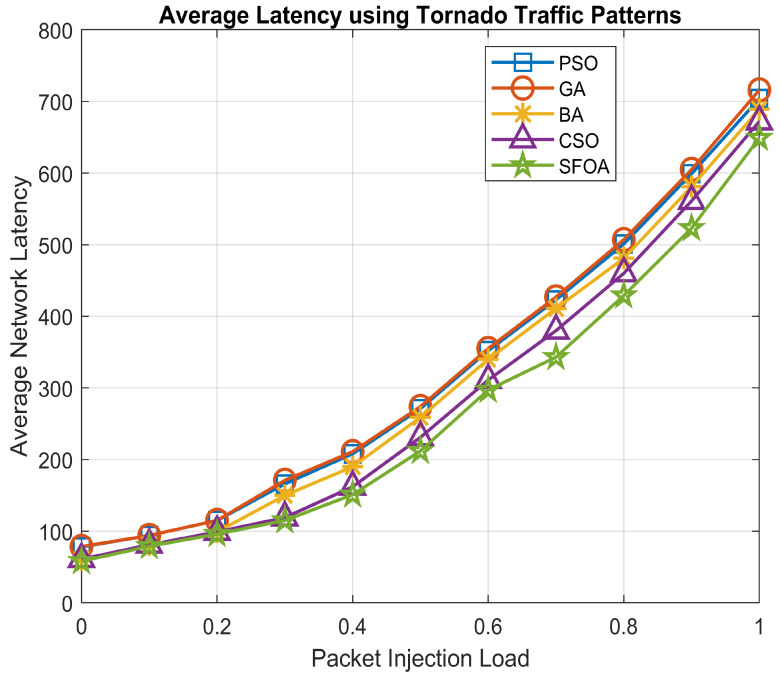
Average network latency for Tornado traffic patterns.

Figure 7 depicts the graphical results of the average network latency in contrast to different rates of injection load under uniform random traffic patterns. It is evident from this graph that SFOA outperformed PSO, GA, BA and CSO by 11.23%, 16.40%, 8.65% and 4.42%, respectively, for uniform random traffic pattern. Furthermore, Figure 8 illustrates the graphical results of the average network latency compared to different injection load rates under tornado traffic patterns. It can be seen from this graph that SFOA outperformed PSO, GA, BA and CSO by 24.06%, 25.45%, 13.89% and 5.82%, respectively, for tornado traffic patterns.

SFOA gives the best latency in comparison with other existing nature-inspired algorithms considered such as PSO, GA, BA and CSO using minimum hops count mapping technique.

The mapping results of the proposed SFOA clearly indicate that it is more efficient than other existing nature-inspired algorithms. The results in figures and tables show the improvement in performance analysis parameters. It indicates the reduction in average network power consumption, computation time, communication cost and average network latency.

## 8. Conclusions

This paper presents a state-of-art nature-inspired metaheuristic algorithm, i.e., SFOA, which mainly comprises two advantages. The first advantage is high-speed convergence by strengthening the searching process used for the best sailfish group. The second advantage is robust optimization by strengthening the search space intended for the diversity of the sardine population. SFOA is used for the optimized mapping of the application task graph on a two-dimensional NoC with mesh topology. The efficiency of the proposed approach was assessed based on the results of the performance analysis parameters for six standard NoC benchmarks. The evaluation of the proposed SFOA proficiency was done via multiple experiments on alternative heuristic algorithms such as ACO, PSO, SA, GA, BA and CSO. The results shown in the previous section indicate that the average improvement of power minimization of the proposed algorithm SFOA is 3.63%, 23.7%, 18.70%, 22.14%, 27.25%, 18.66%, 12.08% and 4.73% over ILP, ACO, PSO, SAT, SA, GA, BA and CSO, respectively. In contrast to other existing mapping techniques, the proposed SFOA takes 69% less computation time. It is evident from the average network latency graphs that SFOA outperformed PSO, GA, BA and CSO for two distinct standards of traffic patterns for NoC by 11.23%, 16.40%, 8.65% and 4.42% for uniform random traffic patterns and 24.06%, 25.45%, 13.89% and 5.82% for tornado traffic patterns, respectively. The experiments results reveal that SFOA outperformed other nature-inspired algorithms to minimize power consumption, computation time, communication cost and latency. Moreover, this work can be continued in various ways, e.g., some hybrid algorithms can be introduced to reduce computation time further. This algorithm can also be implemented on 2D and 3D NoC architectures with different topologies.

## Figures and Tables

**Table 1:** Standard NoC benchmarks details with mesh sizes.

Benchmark	Nodes	Edges	2D Mesh Size
**VOPD [35]**	16	21	4×4
**MPEG4 [31]**	12	26	4×4
**MWD [35]**	12	13	4×4
**MP3encMP3dec [31]**	13	14	4×4
**263encMP3dec [31]**	12	12	4×4
**263decMP3dec [31]**	14	15	4×4

**Table 2:** Simulation environment description.

Network Type	2D Mesh
**Type of Platform**	EMBEDDED
**Embedded applications**	VOPD, MPEG4, MWD, MP3encMP3dec, 263encMP3dec, 263decMP3dec
**Mapping algorithm**	SFOA, CSO, ACO, PSO, SA
**Type of Router**	WORMHOLE-PIPELINE
**Routing algorithm**	XY DIMENSION-ORDERED
**Arbitration Policy**	VIRTUAL CHANNEL ARBITRATION
**Packet delivery type**	WITHOUT ACK
**Packet distribution**	EXPONENTIAL
**Sending ACK policy**	SEND ACK OPTIMALLY
**Packet length (fixed)**	10 (flits)
**Injection rate (flit)**	0.1 (flits/cycle/node)
**Output channel selection**	XY-ORDERED
**Buffer size**	8 (flits)
**Inter-route link length**	10,000 (µm)
**Pipeline type**	8
**Pipeline stages**	4
**Input clock frequency**	1000 (MHz)
**Operating clock frequency**	1000 (MHz)
**Warm-up time**	20,000 cycles

**Table 3:** Total Power (W) for 2D NoC 4×4 Mesh for standard NoC benchmarks.

Mapping Algorithm	VOPD	MPEG4	MWD	MP3encMP3dec	263encMP3dec	263decMP3dec
**ILP**	1.528	1.137	1.012	1.228	1.286	1.211
**ACO**	1.920	1.423	1.218	1.498	1.599	1.738
**PSO**	1.841	1.357	1.112	1.507	1.445	1.561
**SAT**	1.856	1.370	1.236	1.524	1.563	1.624
**SA**	1.971	1.478	1.256	1.590	1.697	1.877
**GA**	1.843	1.356	1.109	1.507	1.445	1.561
**BA**	1.634	1.247	1.110	1.486	1.323	1.313
**CSO**	1.518	1.219	1.023	1.228	1.286	1.198
**Proposed Algorithm**	1.311	1.201	1.015	1.228	1.286	1.148

**Table 4:** Estimation of communication cost for 2D NoC.

Mapping Algorithm	Communication Cost (Hops × Bandwidth) in MB/s	
	**VOPD**	**MPEG4**
**ILP [8]**	4119	3567
**ACO [33]**	-	3633
**PSO [34]**	4119	3567
**SA [32]**	4231	3567
**GA [21]**	4218	3772
**BA [45]**	4119	3567
**CSO [44]**	4119	3567
**Proposed Algorithm**	4119	3567

**Table 5:** Percentage deviation over ILP based mapping techniques.

Mapping Algorithm	Percentage of Communication Cost Deviation	
	**VOPD**	**MPEG4**
**ACO**	-	1.9
**PSO**	0.0	0.0
**SA**	2.7	-
**GA**	2.4	5.7
**BA**	0.0	0.0
**CSO**	0.0	0.0
**Proposed Algorithm**	0.0	0.0

**Table 6:** Communication cost and computation time of 2D NoC 4×4 mesh for standard NoC benchmarks.

Mapping Algorithm	VOPD		MPEG4		MWD	
	**Communication Cost (Hops × Bandwidth) in MB/s**	**Computation Time in Seconds**	**Communication Cost (Hops × Bandwidth) in MB/s**	**Computation Time in Seconds**	**Communication Cost (Hops × Bandwidth) in MB/s**	**Computation Time in Seconds**
**ILP**	4119	4679.341	3567	22.340	1120	210.021
**ACO**	-	-	3633	18.652	-	-
**PSO**	4119	3.785	3567	3.465	1120	3.432
**SA**	4231	3878.527	3567	-	1451	197.541
**GA**	4218	3.925	3772	3.234	1321	3.420
**BA**	4119	2.231	3567	2.925	1122	2.894
**CSO**	4119	2.231	3567	2.010	1122	1.996
**Proposed Algorithm**	4119	1.98	3567	1.96	1120	1.886
**Mapping Algorithm**	**MP3encMP3dec**		**263encMP3dec**		**263decMP3dec**	
	**Communication Cost (hops × bandwidth) in MB/s**	**Computation Time in Seconds**	**Communication Cost (hops × bandwidth) in MB/s**	**Computation Time in Seconds**	**Communication Cost (hops × bandwidth) in MB/s**	**Computation Time in Seconds**
**ILP**	17.021	1435.012	230.407	193.035	19.823	4897.210
**ACO**	17.231	1196.856	-	-	-	-
**PSO**	17.021	3.194	230.407	3.185	19.823	3.188
**GA**	17.133	3.194	230.698	3.185	19.911	3.174
**BA**	17.834	2.653	231.450	2.345	19.936	2.350
**CSO**	17.021	1.785	230.407	1.527	19.823	1.511
**Proposed Algorithm**	17.021	1.585	230.407	1.227	19.823	1.011

## Data Availability

Not applicable.
